# Regional Differences in Epidemiological and Clinical Characteristics, Treatment, and Clinical Outcomes of COVID-19 in Wuhan and Remote Areas of Hubei Province

**DOI:** 10.3389/fmed.2021.667623

**Published:** 2021-07-15

**Authors:** Honggang Ren, Xingyi Guo, Antonio Palazón-Bru, Pengcheng Yang, Nan Huo, Runsheng Wang, Yu Sun, Qinyong Hu, Hua Yang, Guogang Xu

**Affiliations:** ^1^Department of Internal Medicine, Tongji Medical College, Huazhong University of Science and Technology, Wuhan, China; ^2^Vanderbilt Epidemiology Center, Vanderbilt University Medical Center, Nashville, TN, United States; ^3^Department of Clinical Medicine, Miguel Hernández University of Elche, Elche, Spain; ^4^Renmin Hospital of Wuhan University, Wuhan, China; ^5^Division of Epidemiology, Department of Health Sciences Research, Mayo Clinic, Rochester, MN, United States; ^6^Second Medical Center, People's Liberation Army General Hospital, Beijing, China; ^7^Department of Otorhinolaryngology, Union Hospital, Tongji Medical College, Huazhong University of Science and Technology, Wuhan, China; ^8^Cancer Centre, Renmin Hospital of Wuhan University, Wuhan, China; ^9^Department of Respiratory Medicine, Minzu University Hospital of Hubei Minzu University, Enshi, China; ^10^The Second Medical Centre and National Clinical Research Centre for Geriatric Diseases, Chinese People's Liberation Army of China General Hospital, Beijing, China

**Keywords:** SARS-CoV-2, COVID-19, regional differences, mortality, clinical characteristics

## Abstract

**Background:** The Coronavirus disease 2019 (COVID-19) pandemic has been a major threat to global health. Regional differences in epidemiological and clinical characteristics, treatment and outcomes of patients have not yet been investigated. This study was conducted to investigate these differences amongCOVID-19 patients in Hubei Province, China.

**Methods:** This retrospective cross-sectional study analyzed data on 289 COVID-19 patients from designated hospitals in three regions:Urban (Wuhan Union West Hospital), Suburban areas of Wuhan (Hannan Hospital) and Enshi city, between February 8 and 20, 2020. The final date of follow-up was December 14th, 2020. The outcomes were case fatality rate and epidemiological and clinical data.

**Results:** Urban Wuhan experienced a significantly higher case fatality rate (21.5%) than suburban Wuhan (5.23%) and rural area of Enshi (3.51%). Urban Wuhan had a higher proportion of patients on mechanical ventilation (24.05%) than suburban Wuhan (0%) and rural Enshi (3.57%). Treatment with glucocorticoids was equivalent in urban and suburban Wuhan (46.84 and 45.75%, respectively) and higher than Enshi (25.00%). Urban Wuhan had a higher proportion of patients with abnormal tests including liver function and serum electrolytes and a higher rate of pneumonia (*p* < 0.01 for all). Urban Wuhan also had a higher incidence of respiratory failure, heart disease, liver disease and shock, compared with the other two regions (all *p* < 0.05).

**Conclusions:** Our findings revealed that there are regional differences in COVID-19. These findings provide novel insights into the distribution of appropriate resources for the prevention, control and treatment of COVID-19 for the global community.

## Key Messages

- Previous studies have analyzed clinical and demographic characteristics of COVID-19 patients, but differences amongst regions in the same province have not been conducted.
- Differences in epidemiological and clinical characteristics, treatment and outcomes were found to be associated with the different regions in Hubei Province.
- Detailed analysis on the regional differences in mortality, morbidity, clinical and laboratory parameters will be helpful toward understanding of the epidemiology and pathogenesis of COVID-19.

## Introduction

COVID-19 is a newly recognized infectious outbreak first reported in Wuhan, Hubei province in south central China. The disease then experienced a spread first in Hubei province prior to a large super-spreading event throughout China. While the majority of cases in China were confined in Hubei province, the virus has since spread globally, leading to the World Health Organization (WHO) declaring COVID-19 a pandemic on March 11, 2020 (1, 2).

COVID-19 is caused by a new type of coronavirus (SARS-CoV-2) (3, 4), to which the population lacks immunity and is generally susceptible. COVID-19 continues to spread throughout the globe and is highly contagious, affecting all populations, punctuated by family clusters and hospital outbreaks. Severe cases can result in acute respiratory distress syndrome (ARDS) (5, 6), multiple organ dysfunction syndrome (MODS) (7, 8) and death (9).

As of May 4th, 2020, the COVID-19 outbreak has led to 82,880 infected cases and 4,633 deaths in China, and over 3,599,410 cases and 249,754 deaths globally. Increasing evidence highlights the variable clinical presentation of COVID-19, which ranges from no symptoms to potentially deadly pneumonia that is complicated by multi-organ failure. Huang et al. (10) first reported 41 cases of COVID-19 in which most patients had a history of exposure to the Huanan Seafood Wholesale Market. Xu et al. (3) found that, as of early February 2020, patients from Zhejiang province had relatively mild symptoms compared with patients initially infected with SARS-Cov-2 in Wuhan. Yang's study of critically ill patients in Wuhan showed that the mortality of critically ill patients with SARS-CoV-2 infection is extremely high (9). The difference in severity in patients from different regions is intriguing and requires further study.

Among the different core regions in Hubei province of China, patients with COVID-19 were observed to have discordant epidemiological characteristics even though the populations share the same viral clade. However, adequate data on the disparity between regions are lacking.

In this study, we investigated the epidemiology and clinical characteristics of COVID-19 patients and compared these among the three core epidemic regions in Wuhan and remote areas in Hubei province. Our data was obtained from 289 patients in the first multi-center repository of COVID-19 constructed. We also investigated possible risk factors associated with the mortality of the COVID-19. We explored comprehensive estimates of epidemiologic comparative variables of interest, and we also illuminate potential explanations for the observed discrepancies of COVID-19 in Wuhan and remote areas, highlighting strategies related to its containment.

### Geography

Urban Wuhan – the data is derived from patients admitted to Wuhan Union West Hospital. Wuhan is the largest city in central China, with a population of 11.90 million, an area of 3,280 square miles (8396.8 square kilometers) and a population density of ~1,200 people per square kilometer. The population density is based on the entire city of Wuhan, so the density in the vicinity of the hospital, which is in an urban environment, could be much higher. Wuhan Union West Hospital is a 2,000 bed tertiary care center which was a designated hospital for COVID-19 patients.

Suburban Wuhan - Hannan Hospital in suburban Wuhan is a hospital designated for mild to moderate cases of COVID-19. It is situated in a region where the population density is likely to be much lower than urban Wuhan.

Rural Hubei province – The Enshi hospital system is for local COVID-19 patients. The population of Enshi at the county level is ~857,000 people. The area of Enshi occupies 1,534 square miles or 3,972 square kilometers and sits at an elevation of 420 m (1,380 feet). Compared to the super-city of Wuhan, Enshi is a much smaller city built within a mountainous area of Hubei province. Enshi is composed of 8 regions. Patients were admitted to local hospitals but ultimately transferred to Hubei Minzu University Hospital. The hospitals in Enshi are designated for patients with lower disease severity. The overall population density of Enshi is much less than urban Wuhan at about 190 persons per square kilometer, although about 260,700 or 1/3 of the population is concentrated in the urban area of Enshi.

## Methods

### Study Design and Participants

This was a retrospective cross-sectional study of patients from three hospitals or hospital systems, Wuhan Union West Hospital in urban Wuhan, Hannan Hospital in suburban Wuhan, and the hospital system in Enshi, a city in a more rural, mountainous region of Hubei province about 325 miles from Wuhan. Subjects provided oral informed consent and the institutional review boards of all participating institutions approved the study protocols.

### Data Sources

Our multi-center study accessed the medical records of patients with COVID-19 in urban Wuhan (*n* = 79), suburban Wuhan (*n* = 153) and the Enshi hospital district (*n* = 57) between February 8 and March 20, 2020. The final date of follow up was December 14th, 2020. The three regions are graded using standardized criteria as: the large central metro, large fringe metro, small metro (2013 NCHS Urban-Rural Classification Scheme for Counties which is based on the Office of Management and Budget's (OMB) February 2013 delineation of metropolitan statistical areas (MSA) and micropolitan statistical areas (derived according to the 2010 OMB standards for defining these areas) and Vintage 2012 post-censal estimates of the resident U.S. population). This was a retrospective study of patients investigated from February 8 to 20, 2020, at three regions. Inclusion criteria were (*a*) admitted to a region with suspicious symptoms of COVID-19. (*b*) Patient (or legally authorized representative) provides informed consent prior to initiation of any research procedures and agrees to comply with planned research procedures. (*c*) Non-pregnant female or male adult no <18 years of age at time of research enrollment. (*d*) Confirmed laboratory-confirmed SARS-CoV-2 infection as determined by PCR or other commercial or public health assay. (*e*) Illness of any duration, and at least one of the following: Radiographic infiltrates by imaging (chest x-ray, CT scan, etc.), OR SpO2 <=94% on room air, OR requiring supplemental oxygen. Exclusion criteria were patients (*a*) pregnancy or breast feeding; (*b*) anticipated discharge from the hospital or transfer to another hospital which is not a study site within 72 h; (*c*) Allergy to any research medication. Specifically, we reviewed nursing charts, clinical records, laboratory findings, and imaging results for all subjects with a diagnosis of COVID-19 in the three core hospital or hospital systems based on the National Health Commission and CDC, China protocol (the 5th edition). These data have not yet been published.

### Study Outcome

Mortality was measured as a binary variable based on the individuals' death certification. Patients' health services utilization patterns were identified as binary variables (yes/no) of having ventilation, haemodialysis, antibiotic, antifungal, and antiviral treatments.

The following demographic and clinical features were identified from patients' medical records: sociodemographic variables included sex (male and female), race (Han, others), age (continuous variable), and smoking. Parameters of disease severity include the number of symptoms, unilateral or bilateral pneumonia, and imaging characteristics (ground-glass opacification/opacity, yes/no). Comorbidity variables include cardiovascular diseases (hypertension, heart diseases, etc.), chronic kidney disease, chronic liver disease, COPD (chronic obstructive airways disease), diabetes and others. Medical lab tests such as complete blood count, comprehensive metabolic panel (CMP) and clotting factors were also included in our study.

### Statistical Analysis

Continuous variables were summarized as medians. Kruskal-Wallis tests were applied for continuous variables among patients from the different regions. Categorical variables were summarized as counts or percentages. Chi-square tests were applied for categorical variables among patients from different regions. All statistical analyses were implemented using SAS, version 9.4 (SAS Institute, Inc.). A *p*-value < 0.05 for a two-tailed test was considered to be statistically significant.

## Results

### Characteristics of Study Patients

A total of 289 COVID-19 patients admitted to three hospital systems in Wuhan and Enshi were included in the study. The demographic characteristics of the patients are presented in Table 1. The case fatality rate (CFR) was 21.52, 5.23, and 3.51% in urban Wuhan, suburban Wuhan and rural Enshi medical centers, respectively (*p* < 0.05). The selected baseline and demographic characteristics of all patients are shown in Table 1. The median age was 68 years for cases in urban Wuhan, 54 years for cases in suburban Wuhan, and 41 years for cases in Enshi. Demographic variables including smoking status and an exposure history to Huanan Seafood Wholesale Market did not substantially differ among the three regions. No significant differences were found among three regions for coronary heart disease, chronic obstructive pulmonary disease (COPD), chronic kidney disease and chronic liver disease. However, a history of diabetes, hypertension and cerebrovascular diseases and the number of comorbidities were higher among the cases in urban Wuhan than those in suburban Wuhan and Enshi (*p* < 0.05 for all).

**Table 1:** Clinical characteristics of COVID-19 patients from three regions.

**Variables**	**Urban Wuhan (*n* = 79)**	**Suburban Wuhan (*n* = 153)**	**Enshi (*n* = 57)**	***P-*value**^**a**^
Age (year, median [IQR])	68 (15.52)	54 (14.13)	41 (18.43)	<0.001
Gender (male, *n* %)	50 (63.29)	78 (50.98)	34 (59.65)	0.16
Non-smoking (*n*, %)	73 (91.25)	146 (91.82)	49 (89.09)	0.83
Traveled to urban Wuhan (*n*, %)	79 (100)	33 (21.57)	36 (63.16)	<0.001
Huanan Seafood Wholesale Market Exposure (*n*, %)	4 (5.06)	5 (3.14)	1 (1.81)	0.58
Highest temperature (rket E	38.35 (0.95)	38.08 (0.94)	37.40 (3.95)	0.05
Observation days (day, SD)	2.4 (4.45)	2.56 (3.73)	2.38 (3.66)	0.76
**Signs and symptoms (*****n*****, %)**				
Fever	73 (91.25)	144 (90.56)	43 (78.18)	0.03
Fatigue	45 (56.25)	68 (42.77)	20 (36.36)	0.04
Headache	12 (15.19)	7 (4.40)	11 (20.00)	0.001
Dry cough	20 (25.31)	68 (42.77)	16 (20.09)	0.01
Myalgia	14 (17.72)	6(3.77)	4 (7.27)	0.001
Anorexia	36 (45.57)	123 (77.36)	24 (43.64)	<0.001
Dyspnea	36 (45.57)	24 (15.09)	0 (0)	<0.001
Expectoration	24 (30.00)	77 (48.43)	21 (31.18)	0.02
Pharyngalgia	4 (5.00)	19 (11.95)	6 (10.91)	0.23
Diarrhea	10 (12.50)	11 (6.92)	8 (14.55)	0.17
Vomiting	4 (5.00)	14 (8.80)	2 (3.64)	0.32
Number of signs and symptoms (SD)	4.15 (2.23)	4.59 (3.73)	3.58 (1.92)	<0.01
Highest respiratory rate (SD)	22.67 (5.63)	21.47 (7.10)	20.65 (3.18)	<0.01
**Comorbidities (yes**, ***n*****, %)**				
Coronary heart disease	11 (13.75)	12 (7.54)	5 (9.09)	0.31
Diabetes	10 (12.50)	22 (13.83)	0 (0)	0.02
COPD	1 (1.25)	4 (2.52)	0 (0)	0.43
Hypertension	24 (30.00)	38 (23.90)	3 (5.45)	0.002
Cerebrovascular disease	4 (5.00)	0 (0)	0 (0)	0.004
Chronic kidney disease	4 (5)	5 (3.14)	1 (1.81)	0.58
Chronic liver disease	3 (3.75)	1 (0.63)	2 (3.63)	0.18
Others	16 (20.25%)	18 (11.76%)	5 (8.77%)	0.10
Number of comorbidities (SD)	1.59 (1.39)	0.91 (0.94)	0.53 (1.14)	<0.001
**Disease severity**				
General	8 (10.13%)	135 (88.23%)	50 (87.72%)	<0.001
Serious	42 (53.16%)	7 (4.58%)	4 (7.02%)	<0.001
Critical	29 (36.71%)	11 (7.19%)	3 (5.26%)	<0.001

*Values are presented as number (%)*;

a *Relative to urban Wuhan; International normalized ratio; SD, standard deviation; COPD, Chronic obstructive pulmonary disease; APTT, Activated partial prothrombin time*.

### Clinical Symptoms and Signs

Fever was the most common presenting symptom in all three regions. Fever was documented in 91.25% of COVID-19 cases on presentation in urban Wuhan, 90.56% in suburban Wuhan and 78.18% in Enshi. Less common symptoms were fatigue (45 [56.25%]), anorexia (36 [45%]), and dyspnoea (36 [45%]) in urban Wuhan, anorexia (123 [77.36%]) and expectoration (77 [48.43%]) in suburban Wuhan, and anorexia (24 [43.64%]) and fatigue (20 [36.36%]) in Enshi. Dyspnoea was notably absent in COVID-19 cases from Enshi. The frequency of most of the above symptoms generally appeared to be higher in the urban population. Urban Wuhan had a higher rate of fever, fatigue, dry cough, myalgia, anorexia, expectoration, headache, and highest respiratory rate (Table 1). However, the total number of symptoms was higher in suburban Wuhan than the other two areas. No significant differences were found among the three regions for pharyngalgia, diarrhea, and vomiting. Dizziness, rigor, shortness of breath as well as gastrointestinal symptoms, including diarrhea, vomiting, and abdominal pain, were uncommonly found in the core regions. In fact, we carried out the adjustment analysis involving in the socioeconomic and other factors, finally, we found that the results of urban-rural difference in COVID-19 among the three regions remained unchanged.

### Laboratory and Imaging Findings

Laboratory parameters are shown in Table 2. No significant differences were found among urban Wuhan and the other regions for platelet count, prothrombin time, and creatine kinase. However, aspartate aminotransferase (AST), lactate dehydrogenase (LDH), absolute neutrophil count (ANC), C-reactive protein, and blood glucose were higher among patients in urban Wuhan (all *p* < 0.05). Hemoglobin levels in urban Wuhan were lower than in the other two medical centers (*p* < 0.05). More cases in urban Wuhan presented with abnormal blood counts, liver function, and electrolyte disorders that may be related to chronic inflammation than suburban Wuhan and Enshi.

**Table 2:** Laboratory findings and imaging features of COVID-19 patients from three regions.

**Variables**	**Urban Wuhan (*n* = 79)**	**Suburban Wuhan (*n* = 153)**	**Enshi (*n* = 57)**	***P-*value**^**a**^
**Lab tests (SD)**				
White blood cell (×10^9^/L)	8.69 (1.89)	5.27 (3.77)	5.17 (2.81)	0.01
Hemoglobin (g/L)	127.30 (24.18)	132.86 (16.35)	135.12 (19.70)	0.24
Haematocrit (%)	37.65 (6.86)	39.05 (4.39)	48.16 (55.76)	0.08
Platelets (×10^9^/L)	189.89 (107.10)	192.49 (76.83)	174.35 (68.50)	0.19
Neutrophil (%)	73.39 (18.33)	73.59 (45.65)	66.75 (13.53)	0.56
Lymphocyte (%)	19.56 (16.65)	22.12 (12.78)	24.06 (11.17)	0.02
Monocyte (%)	6.51 (3.65)	7.25 (3.11)	8.08 (3.78)	0.11
Eosinophils (%)	0.50 (1.08)	0.57 (0.74)	0.69 (1.01)	0.02
Basophils (%)	0.19 (0.18)	0.15 (0.23)	0.20 (0.18)	0.92
Absolute neutrophil count (×10^9^/L)	5.87 (7.51)	3.71 (2.40)	3.61 (2.68)	<0.001
Absolute lymphocyte count (×10^9^/L)	2.93 (14.56)	0.97 (0.48)	1.12 (0.55)	0.27
Absolute monocyte count (×10^9^/L)	0.56 (1.59)	0.33 (0.15)	0.38 (0.17)	0.23
Absolute eosinophil count (×10^9^/L)	0.16 (1.17)	0.03 (0.09)	0.03 (0.05)	0.68
Absolute basophil count (×10^9^/L)	0.01 (0.02)	0.01 (0.03)	0.01 (0.01)	0.02
C-reactive protein (mg/L)	70.34 (53.09)	58.51 (60.92)	25.99 (38.72)	<0.001
Blood glucose (mmol/L)	8.80 (5.64)	6.31 (1.90)	5.87 (1.27)	<0.001
Albumin (g/L)	32.67 (5.98)	40.58 (4.85)	41.27 (6.39)	<0.001
Total bilirubin (μmol/L)	12.19 (7.67)	8.54 (5.26)	12.39 (5.35)	<0.001
Alanine aminotransferase (U/L)	48.25 (71.48)	28.63 (27.72)	32.06 (53.80)	0.12
Aspartate aminotransferase (U/L)	53.48 (95.87)	34.17 (20.95)	36.61 (32.68)	0.03
Urea nitrogen (μmol/L)	6.17 (3.81)	5.51 (11.00)	4.51 (2.84)	0.59
Creatinine (μmol/L)	78.61 (34.75)	85.49 (123.72)	72.58 (32.06)	0.87
Serum sodium (mmol/L)	136.13 (14.80)	134.32 (4.97)	137.38 (3.11)	0.001
Serum potassium (mmol/L)	3.91 (0.54)	4.14 (3.00)	4.20 (0.54)	0.96
Lactate dehydrogenase (U/L)	408.99 (348.15)	278.33 (140.44)	202.93 (100.21)	<0.001
Creatine kinase (U/L)	140.56 (129.24)	127.30 (159.49)	138.26 (327.09)	0.52
APTT (s)	39.61 (7.07)	35.85 (23.46)	40.14 (7.85)	0.51
Prothrombin time (s)	14.37 (3.29)	14.94 (4.07)	12.87 (1.75)	0.02
L-6, pg/mL	6.1 (1.2)	4.9 (1.0)	4.1 (1.9)	0.12
L-10, pg/mL	4.9 (0.8)	4.7 (0.9)	4.9 (0.7)	0.23
CD4+ T/CD8+ T cell	2.2 (0.5)	2.12 (0.4)	1.9 (0.6)	0.17
**CT imaging features**				
Ground-glass opacity	61 (82.43%)	81 (72.32%)	30 (52.63%)	<0.01
Unilateral pneumonia	6 (8.11%)	16 (10.67%)	14 (24.56%)	0.01
Bilateral pneumonia	67 (90.54%)	133 (88.67%)	32 (56.14%)	<0.001

*Values are presented as number (%)*;

a *Relative to urban Wuhan; International normalized ratio; SD, standard deviation; COPD, Chronic obstructive pulmonary disease; APTT, Activated partial prothrombin time*.

Typical chest computed tomography (CT) and X-ray findings of COVID-19 patients on admission were either bilateral, multiple lobular, or subsegmental areas of consolidation or bilateral ground glass opacity. Compared with the other regions, patients in urban Wuhan displayed a higher frequency of pneumonia and other radiographic abnormalities. In addition to a higher CFR in urban Wuhan, we also observed a higher incidence rate of respiratory failure, heart disease or heart arrhythmia, liver diseases, and shock, compared with other two regions (all *p* < 0.05). Approximately two-thirds of urban Wuhan and half of suburban Wuhan patients had pre-existing comorbid conditions, which was much greater than Enshi (18%, based on available data), and in each region, the mortality was markedly elevated for those patients with comorbidities. When comparing across the three regions, suburban Wuhan and Enshi patients had similar mortality, but CFR was much higher in urban Wuhan.

### Treatment and Disease Outcomes

Compared with the other two regions, COVID-19 patients in urban Wuhan had a higher proportion of ventilator support (both invasive and non-invasive), glucocorticoids and traditional Chinese medicine use. In addition, COVID-19 patients in urban Wuhan had a higher number of observation days than the other two areas (8.56, SD: 5.59 vs. 7.35, SD: 3.97, vs. 4.09, SD: 3.50, *p* < 0.0001, respectively). However, more patients in suburban Wuhan received antibiotic and antiviral treatments (shown in Table 3).

**Table 3:** Treatments and outcomes of COVID-19 patients from three regions.

**Variables**	**Urban Wuhan (*n* = 79)**	**Suburban Wuhan (*n* = 153)**	**Enshi (*n* = 57)**	***P* value**^**a**^
**Treatments**				
Medical observation days (SD)	8.56 (5.59)	7.35 (3.97)	4.09 (3.50)	<0.001
Ventilator support	45 (56.96%)	23 (15.03%)	2 (3.51%)	<0.001
Oxygen therapy	58 (73.42%)	107 (69.93%)	19 (33.33%)	<0.001
Mechanical ventilation (%)	19 (24.05%)	0	2 (3.57%)	<0.001
Invasive	15 (18.99%)	0	1 (1.79%)	<0.001
Non-invasive	18 (22.78%)	1 (0.65%)	2 (3.57%)	<0.001
Renal replacement therapy	7 (8.86%)	0	1 (1.79%)	<0.01
Glucocorticoid therapy	37 (46.84%)	70 (45.75%)	14 (25.00%)	0.02
Immunoglobulin therapy	25 (31.65%)	21 (13.73%)	18 (32.14%)	0.001
Antibiotic therapy	65 (82.28%)	144 (94.12%)	39 (69.64%)	<0.001
Antifungal therapy	9 (11.39%)	0	0	<0.001
Antiviral therapy	69 (87.34%)	147 (96.08%)	51 (91.07%)	0.04
Oseltamivir	8 (10.13%)	27 (17.65%)	5 (8.93%)	0.14
Arbidol	4 (5.06%)	11 (7.19%)	6 (10.71%)	0.46
**Outcomes**				
Respiratory failure	21 (26.58%)	10 (6.54%)	2 (3.51%)	<0.001
Cardiovascular disease (non-arrhythmia)	28 (35.44%)	33 (21.57%)	7 (12.28%)	<0.01
Cardiac arrhythmia	14 (17.72%)	12 (7.84%)	0	<0.01
Liver injury	22 (27.85%)	26 (16.99%)	7 (12.28%)	0.04
Shock	15 (18.99%)	7 (4.58%)	0	<0.001

*Values are presented as number (%)*;

a *Relative to urban Wuhan; International normalized ratio; SD, standard deviation; COPD, Chronic obstructive pulmonary disease; APTT, Activated partial prothrombin time*.

## Discussion

The current analysis of 289 confirmed COVID-19 cases from three centers, two in Wuhan (urban and suburb Wuhan), and the other in Enshi, a more rural area distant from Wuhan but within Hubei province, is presented. Our study included a large sample size across multiple core regions with the same viral clade and a comprehensive assessment that took into consideration epidemiological and clinical features including clinical symptoms, laboratory and imaging findings, intervention and outcomes.

The majority of the results of this study found in our multi-center analyses are generally in line with the results of previous studies (9, 10). Huang et al. (10) first reported 41 cases and found a high rate of multiple organ dysfunction and death in severe cases. Another single-center study by Wang et al. (10) of 138 hospitalized patients with confirmed COVID-19 in Wuhan, China, demonstrated hospital-related transmission of COVID-19. Data from a study conducted in Zhejiang province showed milder clinical characteristics compared to patients from Hubei infected with COVID-19 (3).

Our description of the disparity among different regions in the same province is the first such analysis in the published literature. The most common symptoms at onset of COVID-19 were fever, dry cough, myalgia, fatigue, dyspnoea, and anorexia. Additionally, a significant proportion of patients did not present initially with gastrointestinal symptoms including diarrhea, vomiting, and abdominal pain. Major complications after diagnosis included ARDS, multiple organ dysfunction, shock, and death.

Laboratory and radiological abnormalities were compared across the three groups. Bilateral distribution of patchy shadows and ground glass opacity is a typical hallmark of CT scan findings in COVID-19. Higher AST, lactate dehydrogenase (LDH), and absolute neutrophil count (ANC) levels were seen in patients from urban Wuhan compared to the other two regions. Lymphocyte counts and eosinophil counts were lower in suburban Wuhan and Enshi compared to urban Wuhan, although these were not statistically significant. In addition, a lower hemoglobin level and C-reactive protein were found in patients from urban Wuhan compared to the other two regions. This seems to indicate that both liver and lung injury is worse in urban Wuhan.

The treatment of choice in urban Wuhan was the combined use of antiviral agents, corticosteroids, and intravenous immunoglobulin (11–15). However, there is a scarcity of good scientific evidence for the use of any of these treatments, and randomized controlled trials or well-designed cohort studies are urgently needed to confirm the benefits and risk of each treatment. We observed exceptionally high mortality in the COVID-19 patients with comorbidities in urban Wuhan, and also found similar patterns of mortality for patients with the four most common comorbidities including diabetes, hypertension, cardiovascular disease, and COPD (16, 17). The disparity does not appear to be related to the different levels of general supportive care which included anti-viral or immunologic regimens but may be due to the difference in epidemiological and clinical characteristics of COVID-19 patients among the three regions. Overall, our analysis found that age and pre-existing comorbidity conditions were two major determinants of fatality, which is in line with published studies (18, 19). The reasons for the high mortality among the elderly and those with pre-existing comorbidities remain unclear. This is different from what was observed in the SARS outbreak in 2002–2003 (20).

The large discrepancy in epidemiological and clinical characteristics, treatment and clinical outcomes of COVID-19 across the three regions can only be partly explained by epidemiological and clinical heterogeneities. Our findings underline the importance of a common data collection platform, especially in an emerging epidemic, in order to identify and explain consistencies and differences in the eventual clinical and public health outcomes of infectious disease outbreaks, which is becoming increasingly important in our highly interconnected world.

The covid-19 patients are all admitted to the study hospitals without transfer records. In additionally, although the difference between the medical service levels and education level among the three regions(Wuhan urban is better than Hannan, and then Enshi). But it is true that the disease is worst in Wuhan, followed by the Hannan district on the outskirts of Wuhan city and the worst in Enshi in Hubei province, which is far away from Wuhan. Indeed, the three selected hospitals are representative of the best local hospitals and medical level. Differences in treatment based on the severity of the disease.

It is intriguing to speculate on the reasons for the difference in mortality among the three reasons, in spite of the fact that the viral clade is the same. The geography of the regions of the three regions was presented earlier, as well as population density and elevation. What is not clear is if the lifestyle or dietary differences between the regions play a role in severity or have any effect on immunologic factors such as cytokine release during infection with COVID-19. This would be an interesting area of further research. The population density aspect is particular interesting as well, and the obvious question is whether or not exposure to more infected people (due to closer quarters) leads to a higher viral load, thus leading to higher disease severity. The effect of quarantining in different areas may have had an impact as well, but exactly how this occurs is not clear and would require a finer analysis of quarantine procedures in the three regions.

An interesting result concerned is that the relatively higher infection rate and CFR is also consistent to each subgroup including low risk groups such as non-smoking patients, which indicates that the presence of some potential risk factors not incorporated in our models (e.g., hospital condition and treatment protocols,) may have independently increased infection rate in three regions. We also found similar patterns of CFRs for patients with the seven most common and potential comorbidities (coronary heart disease, diabetes, COPD, hypertension, cerebrovascular disease, chronic kidney disease, and chronic liver disease) between Hannan and Enshi and we did not observe exceptionally high case-fatality ratios among the subjects with these comorbidities in Wuhan urban. Hence the imparity is probably not due to immunologic or anti-viral treatment protocols but could have been associated to different levels of general medical care. Overall, our analysis found that age and pre-existing comorbid conditions were two major determinants of CFRs, which is consistent with existing studies. Male gender was significantly related to increased risk of fatality in Wuhan after adjustment for other important confounding factors, consistent with a previous study that identified a sex effect in unadjusted analyses of aggregate data. The reasons for an increase in risk of death among males remain unclear.

Several limitations of the study should also be acknowledged. Firstly, the retrospective and hospital-based design of this study mean that we are reliant solely on clinically obtained information, and that endpoints were not preselected during study design. Our study is therefore limited by the inevitable missing data and recall bias. Secondly, although the study is a multi-center study of 289 COVID-19 patients, the case sample size for some of the subgroup analyses was relatively small. Thirdly, due to the severity designation of the hospitals, there may be an inherent bias in the type of patients admitted to the three hospitals. Another concern is that the absence of a large number of asymptomatic patients with infectivity and self-reported information for the subjects at baseline may have been affected by preclinical conditions. Clearly one of the most important explanations for the observed regional differences in epidemiological and clinical characteristics, treatment and clinical outcomes of COVID-19 across the three regions in our subset of patients from Wuhan is the inherent selection bias. As previously discussed, the Wuhan patients in our database mostly were hospitalized in Union Hospital and were found to be epidemiologically different from patients in other hospitals, which makes it difficult to generalize our results to all patients in Wuhan, which is the inevitable limitation of our study.

In conclusion, we performed a multi-center comparative analysis model to ascertain the epidemiologic and clinical differences among patients with confirmed COVID-19 in Wuhan and remote areas. The COVID-19 outbreak occurred right around the four day Chinese lunar New Year holiday, during which time there is one of the largest mass movements of people between cities and regions in China (3). Traditional intervention measures such as quarantine and border control in Wuhan since January 2020 were found to be effective in containing the outbreak. Detailed analysis of observations such as those illustrated in this paper on the regional differences in mortality, morbidity, clinical and laboratory parameters will be helpful in the understanding of the pathogenesis and epidemiology of SARS-CoV-2.

## Data Availability Statement

The raw data supporting the conclusions of this article will be made available by the authors, without undue reservation.

## Ethics Statement

This study was approved by the Ethics Committee of Wuhan Hannan Hospital, General Hospital, Wuhan University, Minzu University Hospital, Minzu University, and Wuhan Union West Hospital, Tongji Medical College, Huazhong University of Science and Technology. Patient consent was obtained upon hospitalization according to government policy.

## Author's Note

The lead authors and manuscript's guarantor affirm that the manuscript is an honest, accurate, and transparent account of the study being reported; that no important aspects of the study have been omitted; and that any discrepancies from the study as planned have been explained. Dissemination to participants and related patient and public communities: No study participants were involved in the preparation of this article. The results of the article will be summarized in media press releases from Wuhan Union West Hospital and presented at relevant conferences.

## Author Contributions

QH, HR, HY, and GX conceptualized the paper. HR, XG, and NH analyzed the data with input from RW. The data was collected by QH, YS, and RW. HR and GX wrote the initial draft with all authors providing critical feedback and edits to subsequent revisions. HY and GX are the guarantor. All authors approved the final draft of the manuscript.

## Conflict of Interest

The authors declare that the research was conducted in the absence of any commercial or financial relationships that could be construed as a potential conflict of interest.
